# Proposed Molecular and miRNA Classification of Gastric Cancer

**DOI:** 10.3390/ijms19061683

**Published:** 2018-06-06

**Authors:** Lara Alessandrini, Melissa Manchi, Valli De Re, Riccardo Dolcetti, Vincenzo Canzonieri

**Affiliations:** 1 Pathology, IRCCS CRO National Cancer Institute, 33081 Aviano, Italy; lara.alessandrini@cro.it (L.A.); manchi.melissa@gmail.com (M.M.); 2 Immunopathology and Cancer Biomarkers, IRCCS CRO National Cancer Institute, 33081 Aviano, Italy; 3 The University of Queensland Diamantina Institute, Translational Research Institute, Woolloongabba, QLD 4102, Australia; r.dolcetti@uq.edu.au

**Keywords:** gastric cancer, gene expression profile, gene mutation, molecular gastric cancer subtype, EBV infection, microsatellite, preclinical models, miRNA

## Abstract

Gastric cancer (GC) is a common malignant neoplasm worldwide and one of the main cause of cancer-related deaths. Despite some advances in therapies, long-term survival of patients with advanced disease remains poor. Different types of classification have been used to stratify patients with GC for shaping prognosis and treatment planning. Based on new knowledge of molecular pathways associated with different aspect of GC, new pathogenetic classifications for GC have been and continue to be proposed. These novel classifications create a new paradigm in the definition of cancer biology and allow the identification of relevant GC genomic subsets by using different techniques such as genomic screenings, functional studies and molecular or epigenetic characterization. An improved prognostic classification for GC is essential for the development of a proper therapy for a proper patient population. The aim of this review is to discuss the state-of-the-art on combining histological and molecular classifications of GC to give an overview of the emerging therapeutic possibilities connected to the latest discoveries regarding GC.

## 1. Introduction

Gastric cancer (GC) is the fifth malignant neoplasm worldwide and the third cause of cancer-related deaths [1]. Despite some advances in therapies for GC, long-term survival of patients with advanced disease is poor. GC is a multifactorial disease in which both genetic and environmental factors are involved. Historically, different types of classification have been used to shape prognosis and plan treatment [2,3,4,5,6]. Proposed in 1965, the Laurén system was widely used in GC classification for half a century, which was very useful in evaluating the natural history of GC carcinogenesis. Based on pathological morphology, the Laurén system divides GC into intestinal (G-INT), diffuse (G-DIF) and mixed GC (G-Mix). An improved prognostic classification for GC is essential for the development of a proper therapy for patients. Therefore, based on new knowledge of molecular pathways, new pathogenetic classifications for GC have been proposed. The aim of this review is to update molecular classifications of GC to give an overview of the emerging therapeutic possibilities

## 2. Histological and Molecular Classifications of GC

Based on the gene expression profile for GC cell lines and patients’ tissue, Tan et al. [7] classified GC into two intrinsic genomic subtypes that overlapped with the histological Lauren’s classification. The G-INT subtype and the G-DIF are related to intestinal and diffuse histology, respectively. The two intrinsic subtypes have distinct patterns of gene expression.

In the G-INT subtype, genes associated with the carbohydrate and protein metabolism (FUT2) and cell adhesion (LGALS4, CDH17) are upregulated. The FUT2 gene codes for the galactoside 2-alpha-l-fucosyltransferase 2 enzyme affecting the Lewis blood group involved in *Helicobacter pylori* (*H. pylori*) infection; the LGALS4 gene codes the galectin 4 implicated in the modulation of the interaction between cell-cell and cell-matrix and the peptide transporter cadherin-17 coded by the CDH17 gene.

Instead, in the G-DIF subtype, genes related to cell proliferation (AURKB) and fatty acid metabolism (ELOVL5) are upregulated. The AURKB gene codes for the Aurora B kinase that functions in the attachment of the mitotic spindle to the centromere, and the ELOVL5 gene encodes the elongation of the very long chain fatty acids protein. The prognosis of G-DIF tumour type is poor, and the response to chemotherapy is reduced compared to those of the G-INT type. In vitro, G-INT cell lines are more sensitive to 5-FU and oxaliplatin than G-DIF lines, which result in being more sensitive to cisplatin [7,8]. There were many more other molecular studies based on the Laurén classification [9,10,11,12].

A molecular classification for GC, independent of the histological Laurent classification, was made in 2013 by Singapore Researchers. They categorized GC into three main types: [13] a proliferative profile associated with a high genomic instability and *TP53* gene mutation, a metabolic profile associated with a higher anaerobic glycolysis and resulting in tumour cells more sensitive to 5-FU therapy and a mesenchymal stem cell profile with a high capacity for self-renewal, immunomodulation and tissue regeneration showing a sensitivity to PIK3CA-mTOR pathway inhibitors.

Soon after, The Cancer Genome Atlas (TCGA) research group categorized GC into four main groups by introducing new technologies of large-scale genome sequencing analyses [14]: Epstein-Barr virus (EBV)-positive cancers (9% of all GC) characterized by DNA hypermethylation, a high frequency of PIK3CA mutations and PDL1/PDL2 overexpression, microsatellite instable (MSI, 22%) tumours, showing a very high number of mutations and DNA methylation sites and chromosome instable tumours (CIN, 50%) mainly coding for alteration in tyrosine kinase receptors and genome stable tumours (GS, 20%).

In 2015, by using similar multi-platform molecular approaches, the Asian Cancer Research Group (ACRG) developed a novel molecular classification for GC based on a pre-defined set of genetic pathways relevant to the biology of GC, including epithelial-mesenchymal transition (EMT), microsatellite instability, cytokine signaling and P53 activity [15]. The ACRG classification included four subtypes [16]: an MSI subtype (22.7%), a mesenchymal group microsatellite stable (MSS)/EMT (15.3%) based on the evidence of epithelial-to-mesenchymal transition, a microsatellite stable TP53-positive subtype MSS/TP53+ (26.3%) and a microsatellite stable TP53-negative subtype MSS/TP53− (35.7%), according to the presence/absence of P53 mutations. By using this approach, the MSI subtype had the best prognosis, while the MSS/EMT subtype had the worst one. The former occurred predominantly at an early stage in the distal part of the stomach and showed mainly an intestinal histology (according to Lauren’s classification); the latter occurred at an advanced stage, at a younger age and with a diffuse histology (>80%) including a large set of signet ring cell carcinomas seeding in the peritonea with malignant ascites (64.1% vs. 15–24% in the other subtypes) and showed loss of CDH1 expression. Given the earlier stage of diagnosis, MSI and MSS/TP53− patients also had the best overall survival and when recurrence occurs, this was generally limited to liver metastasis (about 20%). EBV infection was more frequent in the MSS/TP53 active group.

In ACRG, the correlation between molecular classification and prognosis was validated using the TCGA [14] and the Gastric Cancer Project ′08 Singapore datasets [16]. As shown in Table 1, the ACRG subtypes show a significant overlap with the TCGA subtypes, and this confirms the association between better survival and the MSI subtype [17]. However, the overlap is only partial and probably due to the differences in the patient population (Korea in ACRG and USA and Western Europe in TCGA), tumour sampling and technical platforms used. Nonetheless, these novel classifications created a new paradigm in the definition of GC, although some limitations persist:

- these classifications are based on a highly complex methodology, which is not always available in every laboratory;
- they lack a prospective validation on a large scale;
- they have striking differences in epidemiology, underlying molecular mechanisms and prognosis;
- their prognostic power is decreased by limited follow-up of patients;
- none of them takes into account the active, non-malignant stromal cells

## 3. Integrated Molecular Signatures to Discriminate Intestinal and Diffuse Histological GC Subtypes

Previous findings indicated that diffuse and intestinal GC might be two distinct diseases with different molecular bases, aetiologies, epidemiologies and, thus, response to therapies. A recent study based on a population of 300 GC identified 40 genes specifically expressed in diffuse or intestinal GC [12] and three genes associated with the patients’ prognosis, namely EFEMP1 and FRZB in G-DIF and KRT23 in G-INT. The products of the former are an extracellular matrix glycoprotein and a secreted protein regulating bone development and influencing the Wnt/beta-catenin pathway. The latter encodes for a member of the keratin family, which regulates epithelial cell structures.

In the last year, a nine-gene signature, including two negative impact factors (NR1I2 and LGALSL) and seven positive ones (C1ORF198, CST2, LAMP5, FOXS1, CES1P1, MMP7 and COL8A1), was proposed to predict the outcome of GC, and the model was able to predict patients’ outcome in terms of survival and recurrence, clustering GC cases into low-risk and high-risk groups [18].

Although molecular characterizations have identified the gene signature for prognosis in GC, today, signatures are still inadequate for accurate patient therapy. Identifying new tumour markers or constructing gene models is still the focus of many research works and studies.

## 4. TCGA Classification of GC and Related Signaling Pathways

### 4.1. EBV-Related GC

**Figure 1:**
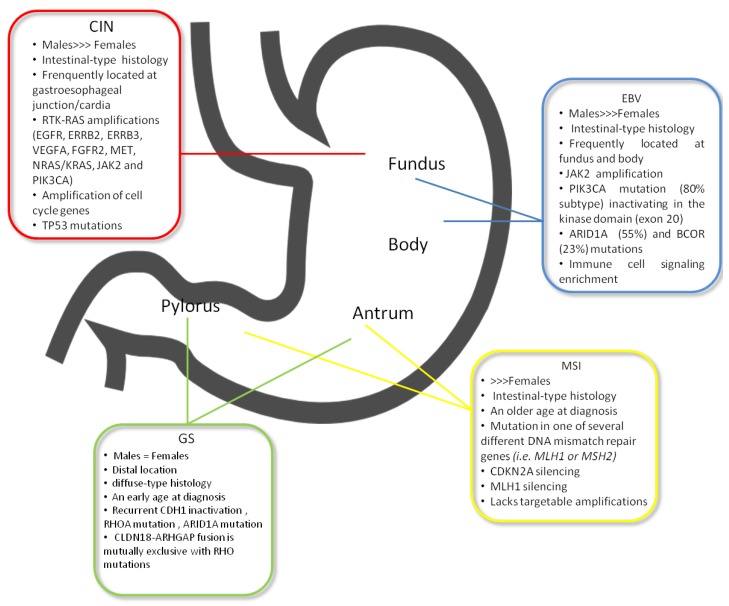
The most relevant clinic-pathological and molecular features of TCGA subtypes.

EBV-positive GC is one of the four subtypes of GC, as defined by TCGA, found in 9% of GC and characterised by high EBV burden [13]. EBV-positive tumours were more frequent in men (81% of the cases) and mainly occurred in the upper part of the stomach. In addition, EBV-positive GC was more prevalent in younger patients compared to older subjects (Figure 1). The histology of EBV-related GC is moderately- to poorly-differentiated adenocarcinoma, often accompanied by dense lymphocytic infiltration [19,20,21,22]. In this subtype were identified pathways related to the elevated expression of programmed death ligands 1 and 2 (PD-L1 and PD-L2), phosphatidylinositol-4,5-bisphosphate 3-kinase, the catalytic subunit α (PIK3CA) mutation and Janus kinase 2 (JAK2) amplification.

PD-L1 helps neoplastic cells to escape from antitumoral immune response, by binding to PD-1, which is expressed on cytotoxic T-cells [23,24,25]. In the literature PD-L1, expressed on cancer cells or tumour infiltrating immune cells, has emerged as a prognostic factor in GC, but its specific role in EBV-related GC has not yet been described [26,27,28,29,30]. In a recent study [31] focusing on EBV-related GC, the expression of PD-1/PDL-1 on immune and neoplastic cells, respectively, was directly related to diffuse histology (according to Lauren’s classification) and depth of tumour invasion. Therefore, targeted immune therapy against the PD-L1/PD-1 axis could be effective in this subtype. Pembrolizumab, a highly specific monoclonal antibody targeting the PD-1 receptor, showed an overall response rate of 22% in a cohort of patients previously treated with chemotherapy [32]. Subsequently, PD-L1 expression in at least 1% of neoplastic cells from paraffin-embedded tissue was significantly related to response to this drug [33]. Another anti-immune strategy, already employed in melanoma, targeting both the PD-1/PD-L1 and the CTLA/B7 axis, is under evaluation in several clinical trials [34].

The PI3K family of intracellular kinases is involved in cell survival, proliferation, differentiation and migration [35]. In GC, the PI3K/AKT/mTOR pathway is frequently activated and associated with nodal metastasis: in 35–80% of GC cases, *PI3KCA* is overexpressed [27,28,29], and in 40–82% of GC cases, phosphorylation of AKT is described [36,37,38,39,40]. The EBV and MSI molecular subtypes of GC show alterations in PIK3CA, in 80% and 42% of cases, respectively [14]. However, molecular mechanisms responsible for sensitivity to PI3K inhibitors are not clearly defined, and the potential use of this drug category in advanced GC is still in the preclinical stage. [41]. In GC, the PIK3CA mutation could be predictive of response to everolimus and AKT inhibitors [42,43]. It is hypothesised that AKT affects the BCL2 protein and the NF-κB pathway. PI3K may also induce upregulation of the chemo-resistance proteins, MDR1/Pgp, BCL2 and XIAP, and downregulation of the expression of BAX and caspase 3. In vitro, in tumour tissues of GC patients, AKT activation and *PTEN* loss were associated with increased resistance to multiple chemotherapeutic agents (5-FU, doxorubicin, mitomycin C and cisplatin) [44]. Similarly, in GC cell lines, a combination of PI3K and AKT inhibitors with chemotherapy agents has successfully attenuated chemotherapeutic resistance [45,46].

The JAK/STAT signaling pathway has been identified in several types of tumours, including GC, and especially in the EBV-subtype [47,48]. The phosphorylation and subsequent activation of JAK2 lead to STAT activation by phosphorylation and activation of downstream gene expression involved in cell proliferation and apoptosis arrest [49]. Therefore, JAK2 inhibitors may also represent a potential therapeutic treatment for solid tumours, such as GC, despite them being primarily studied in inflammatory and myeloproliferative disorders [50]. Ruxolitinib, a JAK1 and JAK2 inhibitor, in combination with capecitabine has demonstrated preliminary efficacy in pancreatic cancer and, in combination with regorafenib, and is currently under evaluation in colorectal cancer (ClinicalTrials.gov identifier: NCI02119676) [51]. However, there are no trials ongoing in GC.

### 4.2. GC with MSI

Microsatellite instability (MSI) is the hallmark of the MSI subtype according to TGCA classification. MSI represents 15–30% of all GCs, is more frequently associated with intestinal histology and usually arises in the mucosa of the antrum, mainly in females at an older age [14,52,53] (Figure 1). MSI is a change that occurs in the DNA of certain cells (such as tumour cells) in which the number of repeats of microsatellites (short, repeated sequences of DNA) is different than the number of repeats in the DNA when it was inherited. The cause of MSI may be a defect in the ability to repair mistakes made when DNA is copied in the cell, determined by mutations in one of several different DNA mismatch repair genes (i.e., *MLH1* or *MSH2*) [54]. The principal mechanism causing MMR deficiency in this GC subtype relies on different *MMR* genes probably involved in MSI-high (MSI-H) sporadic GC without *MLH1* hypermethylation [55,56]. Zhu et al. in a meta-analysis showed a significant reduction of mortality in patients with MSI-H compared with MSI-L (low) or microsatellite stable (MSS) cases [57]. In the MRC MAGIC trial, the relationship between MMRd, MSI and survival in patients with resectable GC randomised to surgery alone or perioperative chemotherapy has been examined. MSI status and *MLH1* deficiency had a positive prognostic role in patients treated with surgery alone, while a negative prognostic effect was established in patients treated with chemotherapy [55]. In contrast to MSI in colorectal cancer, in MSI GC, alterations in *PIK3CA*, *ERBB3, ERB22* and *EGFR* genes, along with major histocompatibility complex I are known [14,53], whereas *BRAF* V600E mutations have never been found [14]. In MSI-positive colorectal cancer, pembrolizumab has shown objective response and progression-free survival rates of 40% and 78%, respectively [58]. Both MSI and EBV subtypes have been associated with a more favourable prognosis and are, therefore, detected in lower percentage in the metastatic setting, with subsequent difficult case finding in clinical trial design [59,60].

### 4.3. GC with CIN

**Figure 2:**
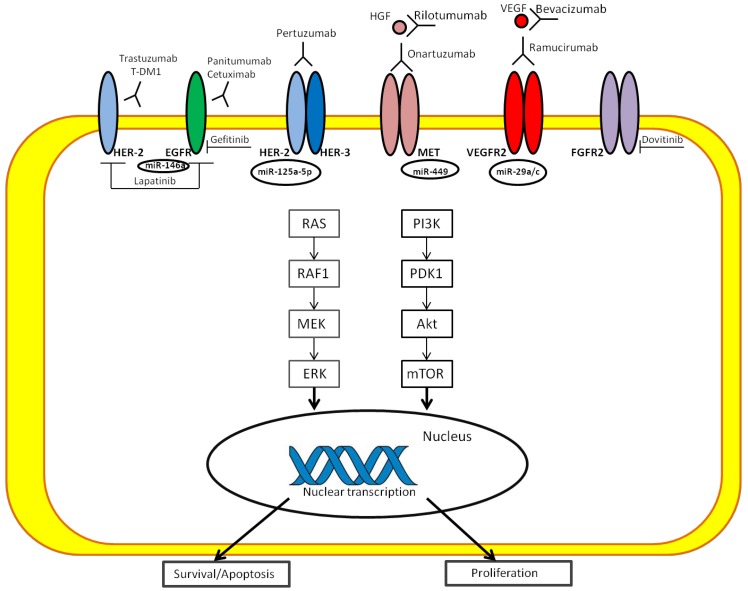
The most relevant targetable pathways in GC.

The largest group, CIN subtype, accounts for approximately 50% of GCs, and its most frequent location is in the esophagogastric junction (EGJ)/cardia, as established by the TCGA study [14] (Figure 1). CIN GC with an intestinal type histology is associated with copy number gains of chromosomes 8q, 17q and 20q, whereas gains at 12q and 13q are more related to diffuse histology [61,62]. The effect of these alterations is the loss or gain of function of oncogenes and tumour suppressor genes [63]. In the CIN subtype, some specific mutations are frequently found, i.e., in the TP53 gene and receptor tyrosine kinases (RTKs), as well as amplifications of cell cycle genes (cyclin E1, cyclin D1 and cyclin-dependent kinase 6) and of the gene that encodes the ligand vascular endothelial growth factor A (VEGFA) [14,64]. Furthermore, HER2, BRAF, epidermal growth factor (EGFR), MET, FGFR2 and RAS mutations have been discovered in the CIN subtype [14,65] (Figure 2).

The most frequent genetic alteration of this subtype, along with their respective targeted drugs, is detailed in Table 2.

### 4.4. Genomic Stable (GS) GC

The GS subgroup included all tumours that did not fulfil appropriate criteria for inclusion in one of the other groups [14]. Patients included in this subgroup represent nearly 20% of all GC, usually show diffuse histology, have a diagnosis at an earlier age (median 59 years), distal localization and occurring equally in males and females (Figure 1). Several subtype-specific molecular changes have been described for GS tumours. The principal somatic genomic alterations observed in GS gastric tumours involve *CDH1*, *ARID1A* and *RHOA* and are described in Table 2. Moreover, an additional translocation (between *CLDN18* and *ARHGAP26*) involved in cell motility was later identified [14].

### 4.5. Patient-Derived Preclinical Models of GC

The lack of effective preclinical models of human tumours, reflecting the complexity and heterogeneity of cancer, has consistently limited the development of targeted drugs. In vitro and in vivo models are available: cancer cell lines; cell line xenograft mouse models (PDX), created transplanting human neoplastic fresh tissue into immunodeficient mice and organoids, which are three-dimensionally cultured tissues, mimicking human tissues [110,111,112,113,114,115,116,117,118,119,120,121,122]. Their advantages and disadvantages are summarised in Table 3.

Stem cell-derived gastric organoids have proven to be effective models of gastric cancer pathogenesis: *H. pylori*-activated c-Met by its virulence factor cytotoxin-associated gene A and induced a two-fold increase in epithelial cell proliferation [123]. Furthermore, epithelial dysplasia was found in gastric organoids, and adenocarcinoma quickly developed in mice having mutations in KRAS or P53 [124]. Murine epithelial-mesenchymal organoids were used also to successfully replicate hereditary GC, with short hairpin RNA knockdown of TGFBR2 [125]. The fundamental role of RHOA function in mediating anoikis in diffuse-type GC was demonstrated also in mouse organoids [126].

### 4.6. Role of microRNAs in Signaling Pathways of GC

MicroRNAs (miRNAs) are short, approximately 22 nucleotides in length that play key roles in the regulation of gene expression [127]. Accumulating evidence indicates that miRNAs play an important role in regulating cancer-related genes. They contribute to GC as oncogenes or tumour suppressors by inhibiting either directly or indirectly the expression of target genes, some of which are involved in signaling pathways [128]. Phosphatase and tensin homologue (PTEN) functions as a tumour suppressor by counteracting PI3K signaling [129]. miRNA-221/222 has been found to be a modulator of PTEN: by antisense or overexpression strategies, it directly affects PTEN expression [130]. PTEN is also a target gene of miRNA-21 that increases the proliferation and invasion of GC cells. A similar effect is displayed by miRNA-214 [131].

miRNA-375 is one of the most downregulated miRNAs in GC, by directly targeting PDK1, a kinase that phosphorylates Akt. Ectopic expression of miRNA-375 reduces cell viability by inducing the caspase-dependent apoptotic pathway [132]. Instead, miRNA-143 regulates the function of GC cells in the PI3K/Akt pathway because its gene target is Akt itself [133]. Down-expression of miR-181c stimulates KRAS expression and may have an important role in GC [134]. It was found that miRNA-29s could influence the Ras/Raf/MEK/ERK pathway, which acts on cell cycle progression by induction of cell cycle regulatory proteins such as CDKs and cyclins. miRNA-29c inhibits protein expression/phosphorylation of Cdc42 [135,136]. Feng et al. demonstrated that CDK6 is regulated by miRNA-107 [137]. Its expression is significantly decreased in GC, and its re-expression significantly decreases proliferation. In GC, miRNA-206 modulates downstream target cyclin D2, involved in proliferation [138]. miRNA-106b and miRNA-93 could be upregulated in GC and be downstream targets of the oncogenic transcription factor E2F1, which make the tumour-suppressive function of transforming growth factor-β less effective [139]. E2F1 is a gene target of miRNA-331-3p and miRNA-106a, modulating the G1/s transition [140,141]. miRNA-331-3p is a tumour suppressor, whereas miRNA-106a promotes tumour growth. A group of researchers demonstrated that the p21 family of CDK inhibitors was suppressed by miRNA-106b-93-25 and miRNA-222-221 clusters. In particular, miRNA-25 targets p57 through the 3′-untranslated region; miRNA-106b and miRNA-93 control p21, whereas p27 and p57 are downregulated by miRNA-222 and miRNA-221 [142]. miRNA-148a has as direct target, p27, so by suppressing p27 expression, it may promote gastric cell proliferation [143]. miRNA-196a, when highly expressed, is associated with clinic-pathological parameters, such as tumour size, poor pT stage, pN stage and patients’ overall survival times. In vitro and in vivo, a downregulation of miRNA-196a suppresses gastric cancer proliferation by targeting p27^kip^ [144]. Previous research has shown that miRNA-375, by targeting the JAK2 oncogene, may act as a tumour suppressor and regulate GC cell proliferation [139]. Moreover, miRNA-135 by targeting JAK2 may repress p-STAT3 activation, reduce cyclin D1 Bcl-xL expression and inhibit cell proliferation [47].

### 4.7. Clinical Implications of Tissue miRNAs in GC

Tissue-based GC-related miRNA biomarkers are listed in Table 4, focusing particularly on their application as diagnostic and prognostic indicators [145,146,147,148,149,150,151,152,153,154,155,156,157,158,159]. Dysregulated expression of miRNA can play an oncogenic or tumour-suppressor role. In fact, they can regulate different signal pathways, targeting genes involved in cell migration, angiogenesis and cell proliferation. Table 5 summarises specific miRNA targeting pathways described above [160,161,162,163,164,165,166,167].

## 5. Conclusions

The recent molecular research on GC has generated large amounts of data that are currently not integrated into clinical practice.

However, they may be of help in the design of future clinical trials aiming to personalise treatment in several ways: (i) by identifying the driving pathways of tumour growth; (ii) by discovering potential drugs targeting such pathways; (iii) by finding predictable mechanisms of resistance and strategies to overcome them.

It must be emphasised that each targetable molecular alteration/pathway is not specific to a distinct subtype of GC; therefore, molecular subgroups alone are not sufficient to assign a patient to a clinical trial. On the contrary, molecular characterization of patients is useful to select a small population to be screened for protocol-eligible molecular aberrations. The implementation of GC research and the molecular classification of patients in clinical trials may be important to select the most appropriate therapies in GC. The hope is that combining histological and molecular classification will be supportive of GC therapeutics and prognosis, but also in the near future for new non-invasive diagnostic approaches such as to identify specific GC biomarker subtypes from circulating nucleic acid or tumour cells.

## Figures and Tables

**Table 1:** Key characteristics of The Cancer Genome Atlas (TCGA) and the Asian Cancer Research Group (ACRG) molecular classifications of gastric cancer (GC). MSI, microsatellite instable; CIN, chromosome instable; GS, genome stable; EGJ, esophagogastric junction; MSS, microsatellite stable.

**TCGA**	**EBV**	**MSI**	**CIN**	**GS**
	- Males >>> Females - Intestinal-type histology - Frequently located at fundus and body - JAK2 amplification - PIK3CA mutation (80% subtype) inactivating in the kinase domain (exon 20) - ARID1A (55%) mutations - Immune cell signaling enrichment	- >>>Females - Intestinal-type histology - An older age at diagnosis - Mutation in one of several different DNA mismatch repair genes (i.e., MLH1 or MSH2) - Lacks targetable amplifications	- Males >>> Females - Intestinal-type histology - Frequently located at EGJ - RTK-RAS amplifications (EGFR, ERRB2, ERRB3, VEGFA, FGFR2, MET, NRAS/KRAS, JAK2 and PIK3CA) - Amplification of cell cycle genes - TP53 mutations	- Males = Females - Distal location - Diffuse-type histology - An early age at diagnosis - Recurrent CDH1 inactivation, RHOA mutation, ARID1A mutation
**ACGR**	**MSS/TP53+**	**MSI**	**MSS/TP53-**	**MSS/EMT**
	- Frequently EBV-positive - Intermediate prognosis - Mutations in ARID1A, APC, KRAS, PIK3CQA and SMAD4	- Distal stomach - Intestinal-type histology - Early stage diagnosis - Favourable prognosis - Hypermutation	- TP53 mutation - Amplification of RTKs - Intermediate prognosis	- Diagnosed at younger age - Diffuse-type histology - Worse prognosis - Low number of mutations

**Table 2:** Gene alteration and their respective targeted drugs.

Gene	Activity/Positivity	Molecular Alteration	Therapeutic Agents	Ref.
HER2	Member of the EGF RTK family Intestinal type (34%), diffuse type (6%) 24% in CIN, 12% in EBV and 7% in MSI subtypes	Amplification Overexpression	Trastuzumab + traditional chemotherapy (ToGA trial) Other anti-HER2 agents (lapatinib, pertuzumab and trastuzumab-emtansine) have not shown significant benefit; resistance is under investigation	[66,67,68,69,70,71,72,73,74,75,76,77,78,79,80,81]
EGFR	Member of the EGF RTK family; forms heterodimers with HER2 10% in the CIN molecular subtype	Amplification Overexpression	Panitumumab and cetuximab showed disappointing results in two large phase III trials; erlotinib and gefitinib were not effective	[82,83,84,85]
MET	RTK family; interacts with HGF 8% in the CIN molecular subtype	Amplification Overexpression	Rilotumumab was associated with significantly longer PFS and OS when added to chemotherapy in treatment-naive molecularly unselected patients with advanced GC; another anti-MET antibody, onartuzumab, did not show any advantage in combination with mFOLFOX	[86,87,88,89,90]
VEGF	Factors of angiogenesis 54–90% of GCs	Overexpression	Bevacizumab (AVAGAST trial) did not show increased OS Ramucirumab (RAINBOW trial) + paclitaxel confirmed OS advantage in a non-Asian population	[91,92,93,94,95,96,97,98]
FGFR	Fibroblast growth factor receptor family 9% CIN molecular subtype	Amplification	A phase II randomised trial is evaluating the activity of AZD4547, an inhibitor of FGFR 1–2 and 3, compared to paclitaxel in second-line treatment Other ongoing trials are testing dovitinib in FGFR2 amplified GC patients or in combination with docetaxel	[14,34]
KRAS	RAS GTPase; recruits the cytosolic protein RAF <5 GCs	Mutation codon 12–13	No target therapies are currently approved for this alteration in GC	[99]
CDH1	Tumour suppressor gene; encodes E-cadherin, a cell adhesion molecules 37% of the GS molecular subtype	Mutations, hypermethylation, downregulated expression	Treatments targeting EMT are under study	[100,101,102]
ARID1A	Tumour suppressor gene involved in chromatin remodelling 20% GS molecular subtype	Inactivating mutations	No target therapies are currently approved for this alteration in GC	[103,104]
RHOA	Rho GTPases are intracellular signaling molecules, regulating cytoskeleton organization, cell cycle and cell motility Diffuse type 30% GS molecular subtype	Mutations Interchromosomal translocation (between *CLDN18* and *ARHGAP26*)	A recent trial tested IMAB362, a chimeric IgG1 antibody against CLDN18.2 showing clinical activity in patients with 2+/3+ immunostaining	[105,106,107,108,109]

**Table 3:** Patient-derived preclinical models of GC: advantages and disadvantages.

	Cons	Pros
Cell line xenografts	- monodimensional - no tumour-microenvironment interaction - loss of architecture - genetic modifications	- rapid analysis of drug response - immortal cell lines allow unlimited source of material - low cost, low complexity
PDX models	- limited source of material - high failure rate of engraftment - long time for establishment - expensive - tissue must be rapidly processed	- reliable representation of tumour heterogeneity - includes microenvironment - can predict response to drugs
Organoids	- no tumour-microenvironment interaction	- high level of architectural and physiological similarity to native tissue - intermediate cost, easy to handle - large-scale drug screening

**Table 4:** Diagnostic and prognostic role of tissue-based GC-related miRNAs.

miRNAs	Role	Expression in Tissue	Note	Ref.
miR-21	Diagnostic	Upregulated	Overexpressed miR-21 binds to PDCD4 and can inhibit protein expression; directly related to tumour size, depth of invasion, lymph node metastasis and vascular invasion	[145,146]
miR-21 miR-223 miR-218	Diagnostic	Upregulated Downregulated	-	[147]
miR-31	Diagnostic	Downregulated	-	[148]
miR-32 miR-182 miR-143	Diagnostic	Upregulated	-	[149]
miR-106a	Diagnostic	Upregulated	Level of miR-106a is closely related to tumour size, differentiation degree, lymph node and distant metastasis	[141]
miR-20 miR-150b miR-451	Prognostic	Upregulated Upregulated Downregulated		[150,151]
miR-29	Prognostic	Downregulated	This miRNA is associated with poor prognosis	[152]
miR-106b	Prognostic	Upregulated	This miRNA is associated with poor prognosis	[153]
miR-125a-5p	Prognostic	Downregulated	Multivariate analysis shows that its downregulation is an independent prognostic factor for survival	[154]
miR-206	Prognostic	Downregulated	mRNA-206 is an independent prognostic factor in GC patients	[155]
miR-17-5p miR-21 miR-106a miR-106b miR-7a	Prognostic	Upregulated	-	[142,156]
miR-10b miR-21 miR-223 miR-338 let-7a miR-30a-5p miR-126	Prognostic	-	These seven miRNAs are significantly related to recurrence-free periods and overall survival of patients; an overexpression of miR-223 in primary GC is associated with less survival without metastasis	[157,158]
miR-125b miR-199a miR-100	Prognostic	Upregulated	These miRNAs are associated with progression of GC	[159]
Let-7g miR-433 miR-214	Prognostic	Upregulated Upregulated Downregulated	Levels of these miRNAs are associated with tumour infiltration depth, lymph node metastasis and tumour stage.	[159]

**Table 5:** Expression and deregulation of miRNAs in gastric cancer.

miRNAs	Relative Expression	Target Gene	Cell Function	Ref.
miR-146a	Upregulated	EGFR	Invasion Migration	[160]
miR-449	Upregulated	MET SIRT1 CDK6	Cell proliferation Apoptosis Cell cycle	[161]
miR-29a/c	Downregulated	VEGF	Vascular cell Metastasis Growth	[162]
miR-181c	Upregulated	KRAS NOTCH4	Cell proliferation	[134]
miR-221 miR-222	Upregulated	CDKN1A CDKN1B CDKN1C	Cell Cycle	[142]
miR-200c	Upregulated	CDH RHO	Metastasis Chemoresistance	[163]
miR-150	Upregulated	EGR2	Apoptosis Cell proliferation	[150]
miR-382	Upregulated	PTEN	Angiogenesis	[164]
miR-124	Upregulated	ROCK1	Cell proliferation Invasion	[165]
miR-125a-5p	Upregulated	ERBB2 E2F3	Cell proliferation Metastasis Invasion Migration	[154,166]
miR-145	Downregulated	ETS1	Migration Invasion Angiogenesis	[167]
